# Treadmill training attenuates pyroptosis in rats with cerebral ischemia/reperfusion injury

**DOI:** 10.22038/IJBMS.2022.64668.14231

**Published:** 2022-10

**Authors:** Fang Luo, Mingjin Zhu, Kunkun Lv, Di Sun, Guifen Yang, Guoyuan Pan

**Affiliations:** 1 Tongde Hospital of Zhejiang Province, No. 234, Gucui Road, Hangzhou, Zhejiang, China

**Keywords:** Apoptosis, Cerebral infarction, Exercise, NLR family, Protein, Pyroptosis, Pyrin domain containing 3

## Abstract

**Objective(s):**

Few studies have investigated the mechanism by which exercise training promotes neural repair during rehabilitation after stroke. In this study, we evaluated the neuroprotective effects of exercise training and pyroptosis-associated factors in the penumbra and elucidated the possible mechanisms.

**Materials and Methods:**

Neurological deficits, body weight, and the infarct size were evaluated, and haematoxylin-eosin (HE) staining was performed. Western blotting and immunofluorescence staining were used to assess NOD-like receptor family pyrin domain-containing 3 (NLRP3) and caspase-1 levels. Interleukin-1β (IL-1β) and interleukin-18 (IL-18) levels were assessed by enzyme-linked immunosorbent assay (ELISA). B-cell lymphoma 2 (bcl-2) and bax protein levels were measured by Western blotting, and terminal deoxynucleotidyl transferase dUTP nick-end labelling (TUNEL) staining was used to evaluate apoptotic cells.

**Results:**

Exercise training decreased neurological deficits and the infarct size in MCAO rats Moreover, NLRP3 inflammasome-associated protein levels in the peri-infarct cortex were decreased by exercise training. Exercise training decreased the serum concentrations of IL1β and IL18, upregulated bcl-2, downregulated bax, and reduced the TUNEL index.

**Conclusion:**

Exercise training suppresses NLRP3 inflammasome activity and inhibits pyroptosis to protect against cerebral ischaemic injury. Exercise training can also suppress apoptosis, which may be the target of exercise-induced neuroprotection, thereby reducing brain injury.

## Introduction

Stroke results in neurological dysfunction and neuronal cell death and is known to be one of the leading causes of disability and death (1-3). Ischaemic stroke (IS) in which brain tissue is damaged as a result of reperfusion, is the most common type of stroke, accounting for approximately 80% of all stroke cases (4). The most effective treatment for this disease is restoring the blood supply to the ischaemic area in a timely manner, which can effectively reduce the risk of dysfunction and death (5). However, during the restoration of blood supply, many pathological processes, such as oxidative stress, excitotoxicity, apoptosis, and inflammation, occur, leading to further damage to the central nervous system (CNS)(6). As effective treatments for brain injury after stroke are lacking, new treatments are urgently needed.

Pyroptosis plays a vital role in cerebral ischaemia and is of great concern to clinicians (7, 8). Pyroptosis is a newly discovered type of lytic cell death characterized by cell swelling, rapid plasma membrane rupture and the release of proinflammatory cell contents (6). Pyroptosis is a unique type of lytic cell death, and the NOD-like receptor family pyrin domain-containing 3 (NLRP3) inflammasome plays an important role in the development of pyroptosis (9). The NLRP3 inflammasome is a multiprotein complex that comprises the NLRP3 protein the adaptor apoptosis-associated speck-like protein containing a CARD (ASC) and pro-caspase-1. After the NLRP3 inflammasome is activated by danger signals, pro-caspase-1 is cleaved through an autocatalytic process to produce the active form, i.e., caspase-1 (10). Caspase-1, which controls the maturation of pro-interleukin-β (IL-1β) and pro-interleukin-18 (IL-18), becomes activated, initiating subsequent responses (11). IL-1β is a potent endogenous pyrogen that participates in many immune responses and stimulates a variety of cytokines and chemokines (12, 13). IL-18 induces the secretion of interferon (IFN)-γ, which is important for the enhancement of cell lysis (14, 15).

Exercise training has been shown to have neuroprotective effects against stroke in clinical trials and animal models(16, 17). Exercise training after stroke can help promote neurovascular regeneration and the repair of damaged nerve cells in the infarcted area and penumbra, highlighting the potential role of neuroplasticity in its effect (18-20). Previous research by our group found that treadmill training can regulate programmed cell death, inhibit autophagy, and reduce the level of apoptosis in the ischaemic penumbra (21, 22). However, whether treadmill training affects pyroptosis has not been studied. Therefore, we speculate that treadmill training after ischaemic brain injury can decrease NLRP3 inflammasome activation and the levels of downstream inflammatory factors to regulate pyroptosis.

## Materials and Methods

***Animals and groups***

Sprague–Dawley (SD) rats were used (male, 250-380 g, and 2-3 months old). The rats were housed under a 12:12 h light: dark cycle, and food and water were provided. Animal experiments were approved by the Zhejiang Academy of Traditional Chinese Medicine, China. The research protocol was reviewed by the Laboratory Animal Welfare Ethics Committee of Zhejiang Academy of Traditional Chinese Medicine, edited as: KTSC2021415.

There were 60 rats in the experiment. The rats were randomly divided into 3 groups: (1) sham operation group (S, n=20); (2) MCAO group (M, n=20); and (3) treadmill training + MCAO group (TM, n=20). 

***Focal ischemia model and treadmill training protocol***

An MCAO model was established. The rats were placed on a 37.0 ± 0.5°C heating pad and anaesthetized with 1.5~2.0% isoflurane. The left common carotid artery, the internal carotid artery and the external carotid artery were separated and exposed, and the right internal carotid artery was occluded with a fine silicon-coated surgical nylon monofilament (L3600, Jia Ling Biotechnology Co. Ltd, China). Blood flow was restored after 90 min of occlusion. Sham-operated rats underwent the same procedure except insertion of a nylon monofilament.

For three days before the operation, all rats underwent treadmill training at a speed of 5 m/min for 15 min/day (Litai Biotechnology Co, Ltd, China). The training intensity was 0 slope. After 24 hr of reperfusion, the TM group underwent exercise training on a motorized treadmill at a speed of 10 m/min for 30 min/day for 7 consecutive days. Rats in the S and M groups were placed on a treadmill for the same duration without running.

***Modified neurological severity scoring***

Neurological deficit scores were assessed 1, 3 and 7 days after MCAO by an investigator blinded to the experimental groups. The mNSS scale was used to assess behaviour, with a higher score (0-18 points) indicating more severe neurological damage (Table 1).

***Weight***

The body weights of the rats were measured at 0, 1, 3 and 7 days after MCAO surgery. Body weight was recorded at a fixed time (8 am).

***Brain infarct volume***

The rats were sacrificed after being anaesthetized with 10% sodium pentobarbital (65 mg / kg, IP). Coronal slices were stained with 1% TTC (BCBX0337, Sigma, USA) at 2 mm intervals for 5 min and then fixed in 4% paraformaldehyde overnight. To determine the infarct volume, the infarct area was calculated from photographs using Image-Pro Plus 6.0 and determined by the indirect method, which corrects for oedema, with the following formula: (contralateral hemisphere volume-volume of infarcted tissue in the lesioned hemisphere)/contralateral hemisphere volume.

***Western blotting***

**The rats were sacrificed after being anaesthetized with 10% sodium pentobarbital (**65 mg / kg, IP). Fresh ischaemic penumbra tissue was isolated. The tissue homogenates were then centrifuged at 4°C in RIPA buffer (P0013B, Beyotime, China) containing 1 mmol/l PMSF. The supernatant was collected, and the proteins were separated by gel electrophoresis and transferred to a polyvinylidene fluoride (PVDF) membrane. After blocking for 1 hr in 5% skim milk, the membranes were successively incubated with diluted primary antibodies overnight. The following primary antibodies were used: anti-NLRP3 (DF7438, Affinity, China), anti-caspase-1 (AF5418, Affinity, China), anti-B-cell lymphoma 2 (bcl-2) (AF6139, Affinity, China), anti-bax (AF0120, Affinity, China) and anti-β-actin (AF7018, Affinity, China). Then, the membranes were incubated with diluted secondary antibodies. The protein bands were visualized using a visualization system (Bio-Rad, Hercules, USA). The density of each target protein band was normalized to the density of the β-actin band.

***Tissue preparation***

To prepare frozen sections, the rats were anaesthetized with 10% sodium pentobarbital (65 mg / kg, IP) and perfused with saline followed by 4% paraformaldehyde via the heart. The tissue was dehydrated in 20% sucrose solution and then sectioned with a cryostat. These sections were for terminal deoxynucleotidyl transferase dUTP nick-end labelling (TUNEL) staining and immunofluorescence.

To obtained paraffin-embedded tissues, the rats were perfused and brain tissue was obtained as described above. The brain tissues were dehydrated and fixed by multiple incubations in ethanol and xylene and then embedded in paraffin. These sections were used for haematoxylin-eosin (HE) staining.

***Hematoxylin-eosin staining***

Brain tissues were preserved by embedding them in paraffin and then sectioned with a microtome at a thickness of 10 microns. After HE staining, the sections were visualized using an Olympus BH-2 microscope (Olympus Optical, London, UK) to examine changes in cell morphology.

***Immunofluorescence***

Brain sections were permeabilized (0.3% Triton X-100, 10 min), blocked (5% bovine serum albumin, 1 hr), and stained with the following primary antibodies: anti-NLRP3 (DF7438, Affinity, China) and anti-caspase-1 (AF5418, Affinity, China). After washing in PBS for 5 min three times, they were incubated with secondary antibody for 2 h. Images were captured using a microscope.

***Enzyme-linked immunosorbent assay (ELISA)***

Serum was collected on the 7th day. The levels of IL-1β (BP-E30419, Shanghai Boyun Biotech Co, Ltd, China) and IL-18 (BP-E30650, Shanghai Boyun Biotech Co, Ltd, China) were examined using an ELISA kit. The serum was centrifuged (14,000 rpm, 5 min), and the supernatant was transferred to microplate plates. The samples were incubated with the antibodies. The absorbance was measured at 450 nm using a microplate reader.

***TUNEL staining***

TUNEL staining was performed with an In Situ Cell Death Detection Kit (11684795910, Roche, USA) following the manufacturer’s instructions. The sections were immersed in proteinase K buffer for 10 min, and then the reaction was quenched with 3% H_2_O_2_. The sections were incubated with TUNEL reaction mixture and then stained with DAPI. 

Statistics

All data are expressed as the mean±SD. GraphPad Prism 7 and SPSS 22.0 were used to plot and statistically analyse the data, respectively. The behavioural data and data on the cerebral infarct size and number of positive cells were analyzed by T test. In other experiments requiring comparison with the S group, one-way analysis of variance (ANOVA) was used. *P*<0.05 was considered statistically significant.

## Results

***Behavioural and body weight changes***

The mNSS of the S group was 0 (Table 2). The performance of the rats in the TM group and the M group in the behavioural tests did not differ on the first day after MCAO. The performance of the rats in the TM group was not significantly different from that of the rats in the M group on day 3. The performance of the rats in the TM group was significantly improved compared to that of the rats in the M group on day 7 (*P*<0.01, Table 2).

There was no difference in body weight on the day of MCAO (Table 3). Body weight was significantly reduced in the M group and the TM group compared with the S group on day 1 (*P*<0.001, both). However, body weight did not differ between the TM group and the M group. The same trend was observed on the third and seventh days after MCAO.

***Infarct volume and changes in organizational structure***

**T**he infarct volumes in the M group and the TM group were 26.16±4.02% and 16.41±2.90%, respectively (Figure 2A, B). The cerebral infarct volume was significantly decreased in the TM group compared with the M group on day 7 (*P*<0.05).

HE staining showed that in the S group, cells were arranged regularly, and cell morphology was normal (Figure 2C). In the M group and the TM group, cells exhibited different degrees of damage, showing irregular arrangement, cell body deformation, and nuclear membrane rupture.

***Evidence of pyroptotic mechanisms***

Western blotting indicated that the expression of NLRP3 in the ischaemic penumbra was markedly increased in the M and TM groups compared with the S group (*P*<0.01 and *P*<0.05, respectively; Figure 3A, B). Notably, a significant decrease in NLRP3 protein levels was observed in the TM group compared with the M group (*P*<0.05). The expression of caspase-1 in the M group was lower than that in the S group (*P*<0.05, Figure 3A, C). Caspase-1 protein levels were decreased in the TM group compared with the M group (*P*<0.05).

NLRP3 and caspase-1 levels were quantified using immunofluorescence (Figure 4). On day 7, a significant decrease in the number of NLRP3-positive cells was observed in the TM group compared with the M group (*P*<0.001, Figure 4A, B). However, no difference in the number of caspase-1-positive cells was observed between the M and TM groups (*P*>0.05, Figure 4 C, D).

***Evidence of inflammatory mechanisms***

IL-1β and IL-18 levels were assessed on day 7. IL-1β release was significantly increased in the M and TM groups compared with the S group (*P*<0.01 and *P*<0.05, respectively; Figure 5A). The IL-1β level in the TM group was significantly lower than that in the M group (*P*<0.05).

IL-18 release was increased in the M group and TM group compared to the S group (*P*<0.001 and *P*<0.01, respectively; Figure 5B). The IL-18 level in the TM group was lower than that in the M group (*P*<0.05).

***Evidence for apoptotic mechanisms***

We measured the level of bcl-2, an antiapoptotic protein, and bax, an apoptotic protein. The results indicated that the level of bcl-2 in the penumbra was markedly decreased in the M group compared with the S group (*P*<0.05, Figure 6A, B). Notably, the bcl-2 protein level was increased in the TM group compared with the M group (*P*<0.05). Moreover, a significant increase in bax protein expression was observed in the M and TM groups compared with the S group (*P*<0.001 and *P*<0.05, respectively; Figure 6A, C). Bax protein levels were decreased in the TM group compared with the M group (*P*<0.05).

The distribution and number of apoptotic cells were assessed using TUNEL staining. The apoptotic index in the TM group was lower than that in the M group (*P*<0.001, Figure 6E).

**Table 1:** Modified neurological severity score points

Motor tests	
Raising rat by the tail	3
Flexion of forelimb	1
Flexion of hindlimb	1
Head moved >10° to vertical axis within 30 s	1
Placing rat on the floor (normal=0; maximum=3)	3
Normal walk	0
Inability to walk straight	1
Circling toward the paretic side	2
Fall down to the paretic side	3
Sensory tests	2
Placing test (visual and tactile test)	1
Proprioceptive test (deep sensation, pushing the paw against the table edge to stimulate limb muscles)	1
Beam balance tests (normal=0; maximum=6)	6
Balances with steady posture	0
Grasps side of beam	1
Hugs the beam and one limb falls down from the beam	2
Hugs the beam and two limbs fall down from the beam, or spins on beam (>60 s)	3
Attempts to balance on the beam but falls off (>40 s)	4
Attempts to balance on the beam but falls off (>20 s)	5
Falls off: No attempt to balance or hang on to the beam (<20 s)	6
Reflexes absent and abnormal movements	4
Pinna reflex (head shake when touching the auditory meatus)	1
Corneal reflex (eye blink when lightly touching the cornea with cotton)	1
Startle reflex (motor response to a brief noise from snapping a clipboard paper)	1
Seizures, myoclonus, myodystony	1
Maximum points	18

One point was awarded for inability to perform the tasks or for lack of a tested reflex: 13 18, severe injury; 7 12, moderate injury; 1 6, mild injury

**Table 2:** mNSS scale data

	Day 1	Day 3	Day 7
S	0	0	0
M	5.60±0.84	5.50±0.85	5.30±0.82
TM	5.70±0.94	4.90±0.81	4.30±0.67**

**P<0.01 vs. the MCAO group

mNSS: Modified neurological severity scoring

**Table 3:** Body weight results

	Day 0	Day 1	Day 3	Day 7
S	260.78±6.02	264.19±7.51	289.42±8.60	312.12±11.46
M	265.17±8.49	232.29±10.80^###^	255.98±7.21^###^	287.17±11.26^###^
TM	257.33±8.22	230.26±7.32^###^	252.78±11.91^###^	282.28±14.79^###^

###*P*<0.001 vs. the S group

S: Sham operation group; M: MCAO group; TM: Treadmill training + MCAO group

**Figure 1:**
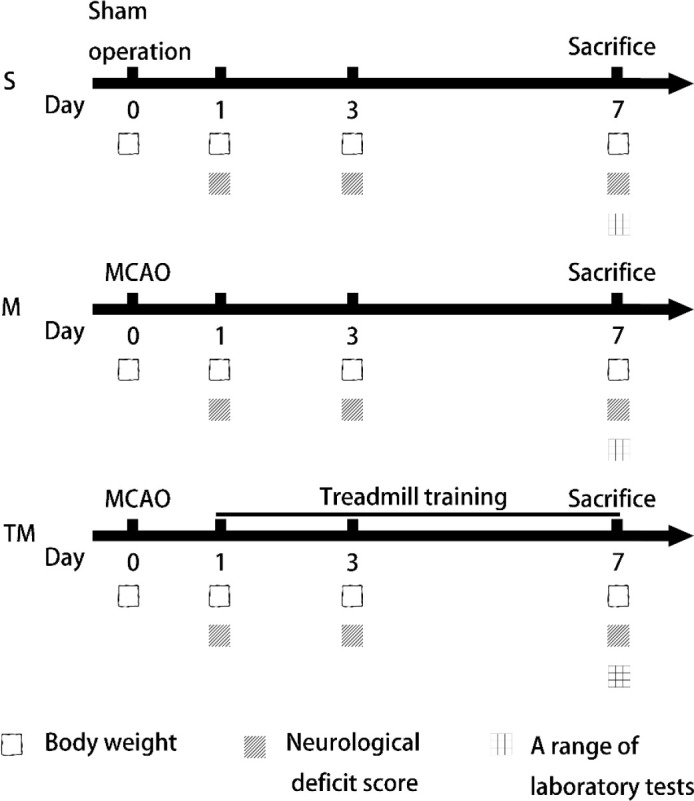
The experimental timeline

**Figure 2:**
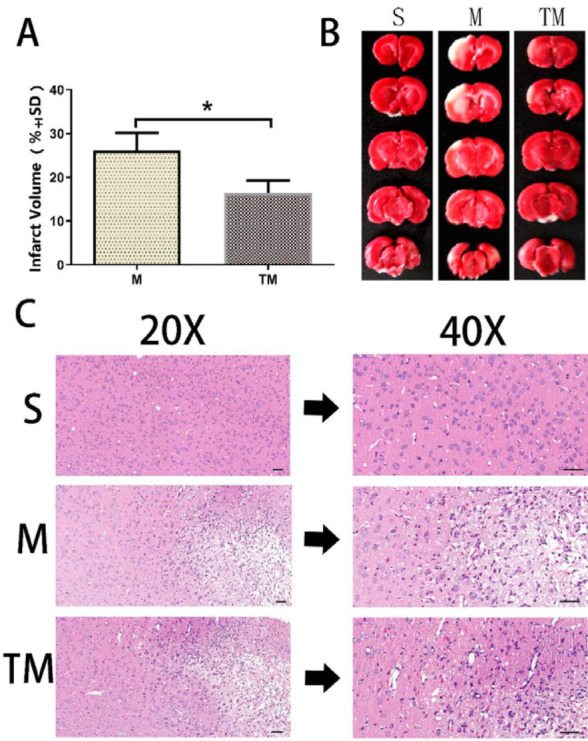
Treadmill training reduced the infarct size and improved organizational structure in rats after MCAO. (A) Quantitative analysis of the infarct size. (B) Representative TTC-stained slices. (C) HE staining. Scale bar, 50 μm. *P<0.05 vs. the MCAO group

S: Sham operation group ; M: MCAO group; TM: Treadmill training + MCAO group HE: Hematoxylin-eosin staining

**Figure 3:**
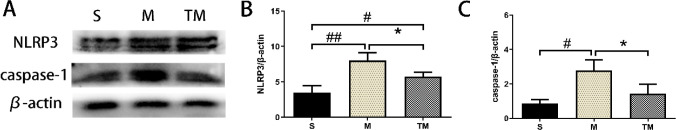
Treadmill training reduced the expression of NLRP3 and caspase-1, as shown by Western blotting. (A) Western blot analysis of NLRP3 and caspase-1 expression. (B) Quantitative analysis of NLRP3 protein expression. (C) Quantitative analysis of caspase-1 protein expression. #*P*<0.05, ##*P*<0.01 vs. the S group. *P<0.05 vs. the MCAO group

S: Sham operation group ; M: MCAO group; TM: Treadmill training + MCAO group

**Figure 4:**
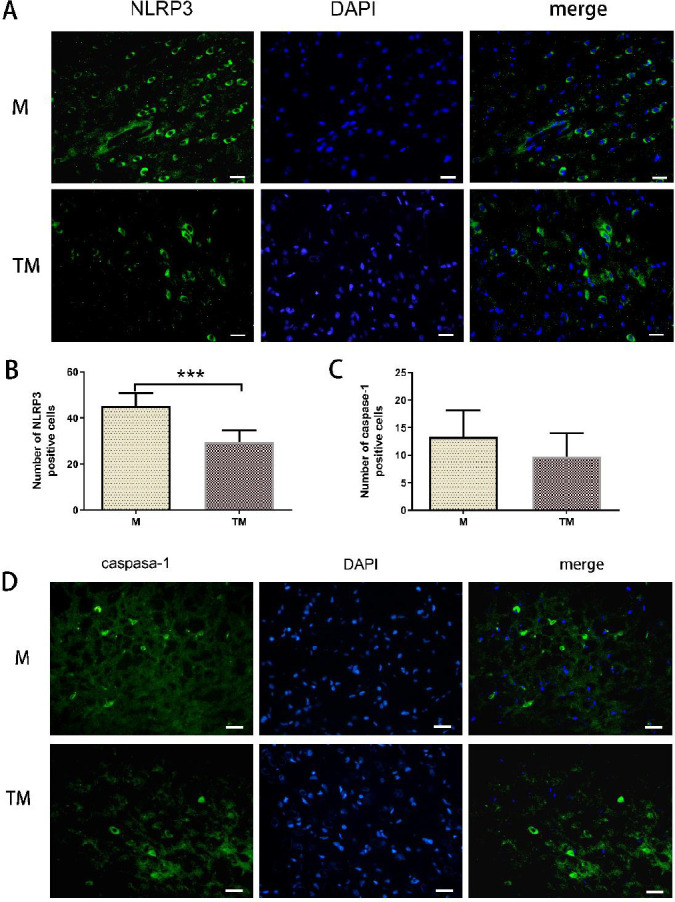
Treadmill training reduced the expression of NLRP3 and caspase-1. (A) Representative images of NLRP3 immunostaining in the penumbra zone. Scale bar, 20 μm. (B) Quantitative analysis of the number of NLRP3-positive cells. (C) Quantitative analysis of the number of caspase-1-positive cells. (D) Representative images of caspase-1 immunostaining in the penumbra zone. Scale bar, 20 μm. ***P<0.001 vs. the MCAO group

S: Sham operation group; M: MCAO group; TM: Treadmill training + MCAO group

**Figure 5:**
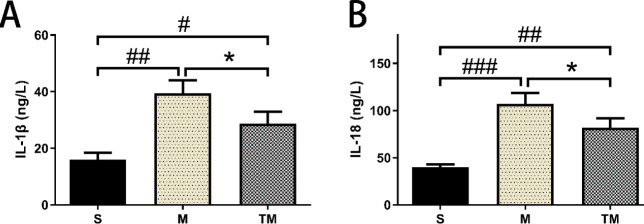
Treadmill training reduced IL-1β and IL-18 release in rats after MCAO. (A) Quantitative analysis of IL-1β levels. (8) Quantitative analysis of IL-18 levels. #*P*< 0.05, ##*P*<0.01 and ###*P*<0.001 vs. the S group. *P< 0.05 and ***P<0.001 vs. the MCAO group

**Figure 6:**
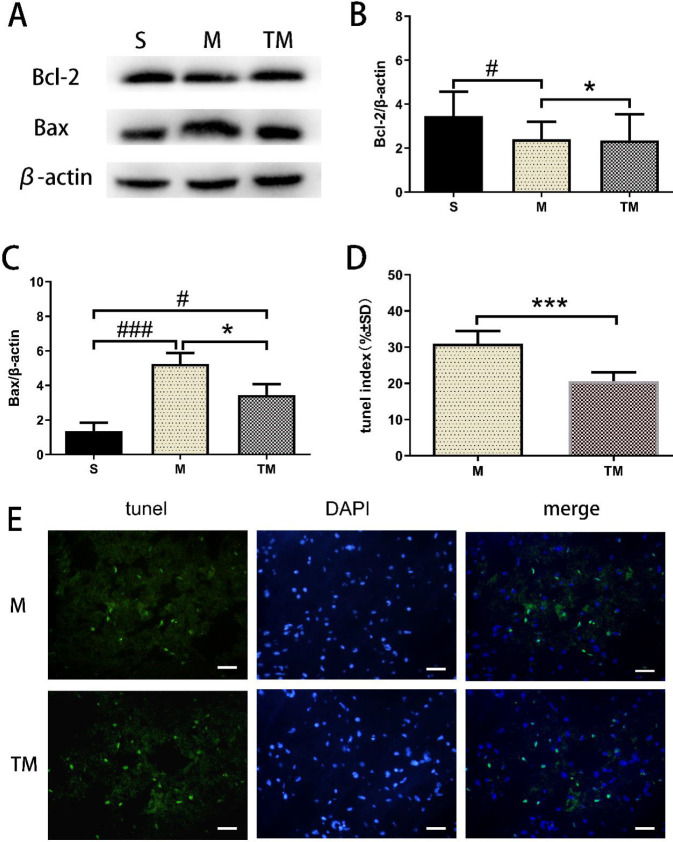
Treadmill training reduced apoptosis in the penumbra. (A) Western blot analysis of bcl-2 and bax levels. (B) Quantitative analysis of bcl-2 protein levels. (C) Quantitative analysis of bax protein levels. (D) Quantitative analysis of the number of TUNEL-positive cells. (E) Representative images of TUNEL staining in the penumbra zone. Scale bar, 20 μm. #*P*<0.05 and ###*P*<0.001 vs. the S group. *P<0.05 and ***P<0.001 vs. the MCAO group

S: Sham operation group; M: MCAO group; TM: Treadmill training + MCAO group

## Discussion

The MCAO model is one of the most commonly used models to simulate IS in humans, as it exhibits both neuromotor deficits and pathological changes (23). In the present study, changes in neurological deficit scores, body weight, and the cerebral infarct volume were monitored, and the results indicated that the MCAO model successfully recapitulated IS. Exercise training has been extensively applied during poststroke rehabilitation (20, 24, 25). Exercise attenuates MCAO-induced neurological deficits and reduces the infarct area, improving neuromotor function and reducing ischaemic brain injury (22, 26). In our experiments, similar findings were obtained. Exercise training reduced the neurological score and infarct volume after IS and exerted a neuroprotective effect.

Pyroptosis involves the activation of the inflammasome, and the role of the NLRP3 inflammasome in the pathophysiological process of stroke has received increasing attention (27). When bound to the adaptor ASC, NLRP3 interacts with pro-Caspase-1 to form the NLRP3 inflammasome. Activated caspase-1 then triggers the maturation of IL-1β and IL-18, initiating corresponding responses (11). Experiments have demonstrated that knockout of NLRP3 inflammasome-related genes can reduce cell death caused by cerebral ischaemic injury (28). In our study, the expression of NLRP3 inflammasome-associated proteins in the penumbra was inhibited by exercise training. In addition, exercise training decreased the serum concentrations of IL1β and IL18. We demonstrated that exercise training exerted a neuroprotective effect by inhibiting pyroptosis. Other studies have provided evidence that exercise training inhibits NLRP3 inflammasome activation in other diseases (Alzheimer’s disease, diabetes, atherosclerosis, etc.) (29-31). Pyroptosis is closely related to inflammation, and it has been suggested that activation of the Toll-like receptor 4 (TLR4)/nuclear factor kappa B (NF-κB) signalling pathway is closely related to NLRP3 inflammasome activation (32). Research has shown that inhibiting NLRP3 inflammasome activation and microglial apoptosis is mediated by suppression of the TLR4/NF-κB signalling pathway (33). However, the exact mechanism remains unclear.

Cells in the brain initiate a variety of death mechanisms after ischaemia (34). Cell apoptosis plays a significant role in IS (11). Bcl-2 is an antiapoptotic protein that promotes cell survival, whereas Bax is a proapoptotic protein that promotes cell death (35). The heterodimer formed by Bax and Bcl-2 is one of the crucial regulatory factors in apoptosis. We found that the expression of Bcl-2 decreased and that the expression of Bax increased in MCAO rats. Exercise training induced the overexpression of Bcl-2 and decreased the expression of Bax. In addition, treadmill exercise was found to reduce the TUNEL index after cerebral ischaemia, showing that exercise training can alleviate cell apoptosis in the penumbra region.

However, some limitations of the current work should be noted. First, the effect of exercise training was only studied at one time point. That is, we only investigated the treatment effect of short-term (1 week) exercise training. In the future, we hope to include later time points and study the effect of more intense exercise. Second, the current study found that exercise may only indirectly regulate NLRP3 and caspase-1. Therefore, the relationship between exercise and pyroptosis needs further to be further investigated, such as by using key molecule inhibitors or knocking out related proteins to directly prove that exercise can inhibit pyroptosis. These topics are worthy of further research.

## Conclusion

Exercise can improve motor function and protect against brain injury. The possible mechanism might involve the regulation of pyroptosis via suppression NLRP3 inflammasome activation, meaning that the NLRP3 inflammation is a potential therapeutic target for stroke. The results of this study provide a new perspective on the mechanism underlying the benefits of exercise therapy.

## Authors’ Contributions

FL and GP Designed the experiments; FL and GP Performed experiments and collected data; MZ and DS Discussed the results and strategy; KL and GY Supervised, directed and managed the study; FL, MZ, DS, KL, GY and GP Final approved of the version to be published.

## Conflicts of Interest

There are no conflicts of interest regarding the publication of this article.
